# Validation of age-specific survival prediction in pediatric patients with blunt trauma using trauma and injury severity score methodology: a ten-year Nationwide observational study

**DOI:** 10.1186/s12873-020-00385-0

**Published:** 2020-11-18

**Authors:** Chiaki Toida, Takashi Muguruma, Masayasu Gakumazawa, Mafumi Shinohara, Takeru Abe, Ichiro Takeuchi, Naoto Morimura

**Affiliations:** 1 Department of Disaster Medical Management, The University of Tokyo, 7-3-1 Hongo, Bunkyo-ku, Tokyo, 113-8655 Japan; 2 Department of Emergency Medicine, Yokohama City University Graduate School of Medicine, 4-57 Urafunecho, Minami-ku, Yokohama, 232-0024 Japan

**Keywords:** Trauma scoring system, Trauma and injury severity score, Survival probability, Children, Japan trauma data bank

## Abstract

**Background:**

In-hospital mortality in trauma patients has decreased recently owing to improved trauma injury prevention systems. However, no study has evaluated the validity of the Trauma and Injury Severity Score (TRISS) in pediatric patients by a detailed classification of patients’ age and injury severity in Japan. This retrospective nationwide study evaluated the validity of TRISS in predicting survival in Japanese pediatric patients with blunt trauma by age and injury severity.

**Methods:**

Data were obtained from the Japan Trauma Data Bank during 2009–2018. The outcomes were as follows: (1) patients’ characteristics and mortality by age groups (neonates/infants aged 0 years, preschool children aged 1–5 years, schoolchildren aged 6–11 years, and adolescents aged 12–18 years), (2) validity of survival probability (Ps) assessed using the TRISS methodology by the four age groups and six Ps-interval groups (0.00–0.25, 0.26–0.50, 0.51–0.75, 0.76–0.90, 0.91–0.95, and 0.96–1.00), and (3) the observed/expected survivor ratio by age- and Ps-interval groups. The validity of TRISS was evaluated by the predictive ability of the TRISS method using the receiver operating characteristic (ROC) curves that present the sensitivity, specificity, positive predictive value, negative predictive value, accuracy, area under the receiver operator characteristic curve (AUC) of TRISS.

**Results:**

In all the age categories considered, the AUC for TRISS demonstrated high performance (0.935, 0.981, 0.979, and 0.977). The AUC for TRISS was 0.865, 0.585, 0.614, 0.585, 0.591, and 0.600 in Ps-interval groups (0.96–1.00), (0.91–0.95), (0.76. − 0.90), (0.51–0.75), (0.26–0.50), and (0.00–0.25), respectively. In all the age categories considered, the observed survivors among patients with Ps interval (0.00–0.25) were 1.5 times or more than the expected survivors calculated using the TRISS method.

**Conclusions:**

The TRISS methodology appears to predict survival accurately in Japanese pediatric patients with blunt trauma; however, there were several problems in adopting the TRISS methodology for younger blunt trauma patients with higher injury severity. In the next step, it may be necessary to develop a simple, high-quality prediction model that is more suitable for pediatric trauma patients than the current TRISS model.

**Supplementary Information:**

The online version contains supplementary material available at 10.1186/s12873-020-00385-0.

## Background

Trauma scoring methods for survival prediction in trauma patients are essential to assess the quality of trauma care because they permit valid comparison of trauma patients who have different anatomical and physiological severities [1]. The Trauma and Injury Severity Score (TRISS) method has been commonly used to calculate the statistical survival probability in trauma patients since its introduction in 1987 by Boyd et al. [2]. After the validation of the revised-version of TRISS by the American College of Surgeons Committee on Trauma coordinated Major Trauma Outcome Study (MTOS) [3, 4] in the Japanese cohort, the TRISS method is reported as a standard technique for estimating survival probability and has commonly been used for evaluating the quality of trauma care [5–8].

The accuracy of the TRISS method, nevertheless, has various challenges in terms of the investigated area, time, and age. First, previous studies suggested that the TRISS has a low accuracy for survival prediction in patients with higher severity of the injury or younger pediatric patients [9, 10]. Second, previous studies suggested that there is a trend to improve the observed-to-expected mortality ratio in major trauma patients, and therefore, new coefficients should be calculated according to these improvements in trauma care for the TRISS to maintain the accuracy for survival prediction [9, 11]. Finally, there are also studies indicating that the modified TRISS methodology with local database-derived coefficients might enhance the accuracy of survival prediction in all regions except the USA, wherein the original TRISS methodology was developed because there are marked differences by region such as in Asian countries [12, 13].

Although the birth rate and mortality of the Japanese population have changed yearly [8, 14], to the best of our knowledge, no study has evaluated the validity of the TRISS method in a pediatric cohort by detailed classification of patients’ age and severity in Japan. Therefore, this study aimed to evaluate the validity of the TRISS method in predicting the survival of Japanese pediatric patients with blunt trauma by detailed classification of age (neonates/infants, preschool children, schoolchildren, and adolescents) and severity of the injury. This study analyzed data obtained from the Japan Trauma Data Bank (JTDB) for the 10-year study period during 2009–2018.

## Methods

### Study design, setting and population

This retrospective, nationwide, observational study analyzed data obtained from the JTDB, which registers data of patients with trauma and/or burn and records prehospitalization and hospital-related information. The JTDB records data of demographics, comorbidities, injury types, mechanism of injury, means of transportation, vital signs, Abbreviated Injury Scale (AIS) score, Injury Severity Score (ISS), prehospital/in-hospital procedures, trauma diagnosis as indicated using the AIS, and clinical outcomes. In most cases, physicians who are trained in AIS coding by using the 1990 revision of AIS [15] undertake the online registration of individual patient data. The JTDB data collection started in 55 hospitals in 2003. The number of participating hospitals in the JTDB registry increased yearly, up to a total of 280 hospitals, including 92% of Japanese government-approved tertiary emergency medical centers in March 2019. The Japan Association for the Surgery of Trauma permits open access and update of existing medical information and the Japan Association for Acute Medicine evaluates the submitted data.

**Fig. 1:**
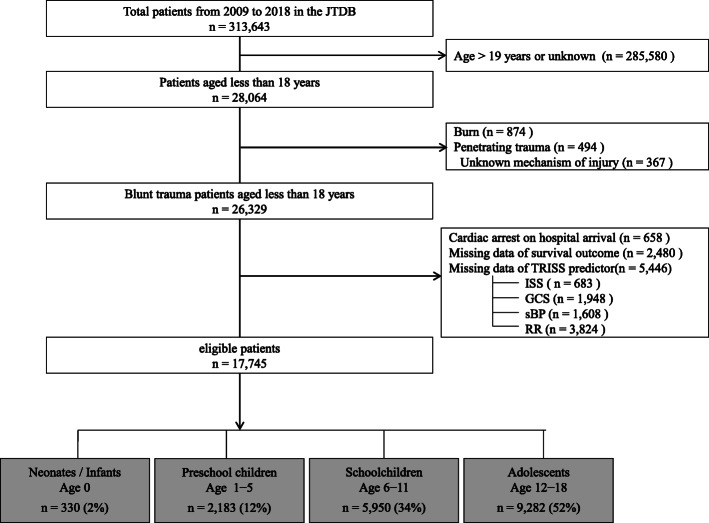
Flow diagram of the study patient disposition

Figure 1 shows a flow diagram of the patient disposition. In this study, we used a JTDB dataset that included information for the period January 1, 2009, to December 31, 2018, which initially yielded the data of 313,643 patients. The inclusion criteria for this study were as follows: the presence of trauma and age 18 years or less. Patients aged 19 years or more, with burns or penetrating trauma, with cardiac arrest on hospital arrival, or with missing data of outcome and TRISS prediction were excluded from this study. Among 26,329 patients with blunt trauma and younger than 18 years, 2480 (9.4%) patients had missing data of survival and 5446 (20.7%) patients had the missing data for TRISS predictor, and hence, the survival probability (Ps) was not calculated using the TRISS method. Furthermore, 683 (2.6%), 1948 (7.4%), 1608 (6.1%), and 3824 (14.5%) patients had missing data of ISS, Glasgow Coma Scale (GCS) score, systolic blood pressure (sBP), and respiratory rate (RR), respectively. Table S1 shows the number of patients who had missing data by age category and each variable.

### Data collection

We collected information on the following variables from the JTDB: age (years), sex, AIS, AIS of the injured region, Revised Trauma Score [3], ISS [10], Ps, and in-hospital mortality. The TRISS ranges from 0 (certain death) to 1 (certain survival), and the survival probability (Ps) is calculated as follows:

$$ \mathrm{TRISS}=\mathrm{Ps}=1/\left(1+{\mathrm{e}}^{\hbox{-} \mathrm{b}}\right) $$

where b = b0 + b1(RTS) + b2(ISS) + b3(age).

RTS is calculated using the GCS score, the sBP, and the RR.

$$ \mathrm{RTS}=0.9368\ast \mathrm{GCS}+0.7326\ast \mathrm{sBP}+0.2908\ast \mathrm{RR} $$

### Data analysis

The outcomes were as follows: (1) patients’ characteristics and mortality by age groups (neonates/infants aged 0 years, preschool children aged 1–5 years, schoolchildren aged 6–11 years, and adolescents aged 12–18 years), (2) validity of Ps assessed using the TRISS methodology by the four age groups and six Ps-interval groups (0.00–0.25, 0.26–0.50, 0.51–0.75, 0.76–0.90, 0.91–0.95, and 0.96–1.00), and (3) the observed/expected survivor ratio by age- and Ps-interval groups. In the primary analysis, which was conducted to identify the characteristics of pediatric trauma patients during the study period, the Mann–Whitney U test and the Kruskal–Wallis test were used for analyzing continuous variables, whereas, a chi-square test was used for analyzing categorical variables. In the secondary analysis, the validity of TRISS was evaluated by the predictive ability of the TRISS method using the receiver operating characteristic (ROC) curves that present the sensitivity, specificity, positive predictive value, negative predictive value, accuracy, area under the receiver operator characteristic curve (AUC), and its 95% confidence interval (CI) of TRISS and show the ability of TRISS to distinguish between positive and negative outcomes. The AUC varies as < 0.7 (low performance), 0.7–0.9 (moderate performance), and > 0.9 (high performance) [16]. In the third analysis, the expected survival calculated using TRISS Ps was compared with the actual Ps. The expected number of survivors in each Ps-interval group was calculated by integrating mean Ps and the number of patients for six Ps-interval group. The results of these comparisons are expressed as the medians and interquartile ranges (IQRs; 25th–75th percentile) for continuous variables and as the mean and percentages for categorical variables. All statistical analyses were performed using STATA/SE software, version 16.0 (StataCorp; College Station, Texas, USA). A two-tailed *P*-value of less than 0.05 indicated statistical significance.

## Results

During the 10-year study period, the data of 17,745 pediatric patients with blunt trauma were included (Fig. 1). The median age and Ps of the total cohort were 13 years (IQR, 8–17) and 0.99 (IQR, 0.98–0.99), respectively. The overall in-hospital mortality rate was 2.1%.

Table 1 shows the demographic and characteristics and variables by age. There were significant differences in all variables by the age category considered, except for neck injury with AIS ≥ 3. Neonates/infants had the highest percentage of head injury with AIS ≥ 3 (88%), highest mean ISS, lowest RTS, and lowest median Ps compared to those of the other age categories.

**Table 1:** Comparison of demographic characteristics and variables by age groups

Variables	Total*n* = 17,745	Neonates / Infants *n* = 330	Preschool children *n* = 2183	School children *n* = 5950	Adolescents *n* = 9282	*P* value
Age, years (median IQR)	13 (8–17)	0 (0–0)	3 (2–4)	9 (7–-11)	16 (15–17)	<.001
Male, n (%)	12,905 (73)	219 (66)	1398 (64)	4264 (72)	7024 (76)	<.001
Injury region, n (%)						
Head injury with AIS ≥ 3	6010 (34)	266 (81)	856 (39)	1987 (33)	2901 (31)	<.001
Facial injury with AIS ≥ 3	173 (1)	0	12 (0.6)	43 (0.7)	118 (1)	<.001
Neck injury with AIS ≥ 3	20 (0.1)	0	3 (0.1)	3 (0.1)	14 (0.2)	0.291
Chest injury with AIS ≥ 3	3339 (19)	10 (3)	349 (16)	862 (14)	2118 (23)	<.001
Abdominal and pelvic injury with AIS ≥ 3	1435 (8)	3 (0.9)	86 (4)	468 (8)	788 (8)	<.001
Spinal injury with AIS ≥ 3	744 (4)	1 (0.3)	26 (1)	85 (1)	632 (7)	<.001
Upper extremity injury with AIS ≥ 3	1610 (9)	0	213 (10)	846 (14)	551 (6)	<.001
Lower extremity injury with AIS ≥ 3	2733 (15)	13 (4)	233 (11)	828 (14)	1659 (18)	<.001
Injury Severity Score, (median IQR)	10 (6–17)	16 (9–17)	9 (5–17)	9 (8–16)	10 (6–19)	<.001
Revised Trauma Score, (median IQR)	7.84 (7.55–7.84)	7.55 (6.61–7.55)	7.55 (6.90–7.84)	7.84 (7.55–7.84)	7.84 (7.55–7.84)	<.001
Survival probability, (median IQR)	0.99 (0.98–0.99)	0.98 (0.95–0.99)	0.99 (0.98–0.99)	0.99 (0.99–0.99)	0.99 (0.98–0.99)	<.001
Survival probability 0.96–1.00, n (%)	15,258 (86)	238 (72)	1882 (86)	5325 (90)	7813 (84)	<.001
Survival probability 0.91–0.95, n (%)	937 (5)	48 (15)	108 (5)	270 (5)	511 (6)	<.001
Survival probability 0.76–0.90, n (%)	844 (5)	29 (9)	116 (5)	214 (4)	485 (5)	<.001
Survival probability 0.51–0.75, n (%)	344 (2)	6 (2)	34 (2)	72 (1)	232 (3)	<.001
Survival probability 0.26–0.50, n (%)	190 (1)	5 (2)	21 (1)	30 (0.5)	134 (1)	<.001
Survival probability 0.00–0.25, n (%)	172 (1)	4 (1)	22 (1)	39 (0.7)	107 (1)	0.022
Mortality, n (%)	378 (2.1)	14 (4.2)	43 (2.0)	73 (1.2)	248 (2.7)	<.001

*AIS* Abbreviated Injury Scale, *IQR* interquartile range

Table 2 shows the accuracy and AUC of TRISS for each age category. In all age categories, the AUC of TRISS demonstrated high performance (0.935, 0.981, 0.979, and 0.977). Table 3 shows the accuracy and AUC of TRISS by each Ps-interval group. The AUC for TRISS was 0.865, 0.585, 0.614, 0.585, 0.591, and 0.600 in Ps-interval groups (0.96–1.00), (0.91–0.95), (0.76. − 0.90), (0.51–0.75), (0.26–0.50), and (0.00–0.25), respectively. The AUC of TRISS demonstrated moderate performance in the Ps-interval (0.96–1.00) group (AUC, 0.865); however, the AUC of TRISS demonstrated low performance in other Ps-interval groups.

**Table 2:** Validation analysis and AUC of the TRISS model by age groups

	No. of patients	Sensitivity,%	Specificity,%	PPV,%	NPV,%	Accuracy, %	AUC, (95% CI)
Total	17,745	99.5	42.3	98.8	64.3	98.3	0.978 (0.973–0.982)
Neonates / Infants	330	99.4	28.6	96.9	66.7	96.4	0.935 (0.858–1.000)
Preschool children	2183	99.3	34.9	98.7	50.0	98.0	0.981 (0.974–0.988)
Schoolchildren	5950	99.7	46.6	99.3	63.0	99.0	0.979 (0.969–0.988)
Adolescents	9282	99.4	43.2	98.5	67.3	97.9	0.977 (0.971–0.983)

*PPV* positive predictive value, *NPV* negative predictive value, *AUC* area under the Receiver Operator Characteristic, *CI* confidence interval

**Table 3:** Validation analysis and AUC of the TRISS model by survival probability interval

	No. of patients	Sensitivity,%	Specificity,%	PPV,%	NPV,%	Accuracy, %	AUC, (95% CI)
Total	17,745	99.5	42.3	98.8	64.3	98.3	0.978 (0.973–0.982)
0.96–1.00	15,258	100.0	0.0	99.9	0.0	99.9	0.865 (0.820–0.911)
0.91–0.95	937	100.0	0.0	98.2	0.0	98.2	0.585 (0.447–0.723)
0.76–0.90	844	100.0	0.0	92.1	0.0	92.1	0.614 (0.546–0.682)
0.51–0.75	344	100.0	0.0	76.7	0.0	76.7	0.585 (0.514–0.655)
0.26–0.50	190	62.7	45.0	61.1	46.8	55.3	0.591 (0.509–0.673)
0.00–0.25	172	0.0	100.0	0.0	72.1	72.1	0.600 (0.611–0.787)

*PPV* positive predictive value, *NPV* negative predictive value, *AUC* area under the Receiver Operator Characteristic, *CI* confidence interval

Table 4 shows the observed-to-expected survivor ratio in the Ps interval by age category. In all the age categories considered, the observed survivors among patients with Ps interval (0.00–0.25) were 1.5 times or more than the expected survivors calculated using the TRISS method.

**Table 4:** Observed-to-expected survivor ratio in each Ps interval by age categories

	Ps interval	Mean Ps	No. of patients	Observed survivors	Expected survivors	Observed / Expected
Total	0.96–1.00	0.991	15,258	15,248	15,121	1.01
	0.91–0.95	0.944	937	920	885	1.04
	0.76–0.90	0.858	844	777	724	1.07
	0.51–0.75	0.646	344	264	222	1.19
	0.26–0.50	0.398	190	110	76	1.45
	0.00–0.25	0.130	172	48	22	2.15
Neonates / Infants	0.96–1.00	0.987	238	237	235	1.01
	0.91–0.95	0.946	48	47	45	1.04
	0.76–0.90	0.864	29	26	25	1.04
	0.51–0.75	0.612	6	3	4	0.82
	0.26–0.50	0.384	5	2	2	1.04
	0.00–0.25	0.163	4	1	1	1.53
Preschool children	0.96–1.00	0.990	1882	1882	1863	1.01
	0.91–0.95	0.944	108	107	102	1.05
	0.76–0.90	0.859	116	103	100	1.03
	0.51–0.75	0.645	34	25	22	1.14
	0.26–0.50	0.391	21	14	8	1.71
	0.00–0.25	0.121	22	9	3	3.38
Schoolchildren	0.96–1.00	0.991	5325	5322	5277	1.01
	0.91–0.95	0.945	270	263	255	1.03
	0.76–0.90	0.859	214	200	184	1.09
	0.51–0.75	0.644	72	62	46	1.34
	0.26–0.50	0.391	30	17	12	1.45
	0.00–0.25	0.131	39	13	5	2.54
Adolescents	0.96–1.00	0.991	7813	7807	7743	1.01
	0.91–0.95	0.943	511	503	482	1.04
	0.76–0.90	0.857	485	448	416	1.08
	0.51–0.75	0.648	232	174	150	1.16
	0.26–0.50	0.401	134	77	54	1.43
	0.00–0.25	0.130	107	25	14	1.80

*Ps* survival probability, *AUC* area under the Receiver Operator Characteristic, *CI* confidence interval

## Discussion

We evaluated the validity of the TRISS method in Japanese pediatric patients with blunt trauma by age-group and severity of injury from the JTDB registry during 2009–2018. This study showed that the performance of the TRISS methodology was lower in the case of survival prediction for pediatric patients with younger age and/or Ps ≤ 0.95, and TRISS underestimated expected survivors in pediatric patients with Ps ≤ 0.25.

Because the accuracy of the TRISS model may reflect the influence of demographic differences in trauma such as the trauma care system or the population structure between the sample area and the USA, wherein the TRISS method was developed, local database-derived coefficients may further enhance the predictive performance of the TRISS [12, 13, 17, 18]. Previous studies based on a Japanese cohort, including children registered in the JTDB during 2005–2008 and 2009–2013 proved that the AUC of TRISS was 0.962 and 0.948 [9, 17]. These Japanese studies focused on pediatric patients with blunt trauma and demonstrated that the TRISS method had a high performance only for Japanese pediatric patients, as in a previous study [9, 18]. Although it is difficult to compare the results between previous studies and this study because of the different periods when the studies were conducted, our results suggest that the TRISS model may be appropriate for Japanese pediatric patients with blunt trauma. However, there is no unified consensus on whether TRISS is a suitable prediction model for pediatric patients. One study recommended the use of the TRISS methodology for both adult and pediatric patients because both TRISS models with and without pediatric coefficients equally predict survival with high performance in pediatric patients with blunt trauma [13, 19]. In the other study, the TRISS model had significantly lower performance than the revised TRISS model based on age-adjusted weights (AUC, 0.785 vs. 0.985, *p* < 0.05)) [10, 20]. Our results suggest several problems in adopting the TRISS model for pediatric blunt trauma patients of all ages or all severity, although our results showed that TRISS had high performance in the overall pediatric cohort.

In this subclass analysis by age category, the accuracy of the TRISS model for neonates/infants was lower than that of the other age categories. First, the neonate/infant group sustained the largest proportion of severe head injury with ISS ≥ 3 in this study. A previous study showed that the accuracy of the TRISS model for pediatric trauma patients with head injury or younger than 5 years was significantly inferior to that for the other pediatric-specific model [10]. Second, the abovementioned finding might be attributed to the higher proportion of patients with a head injury in neonates /infants than the other age-groups in this study cohort (81% vs 31–39%, *P* < 0.001). Finally, another reason was considered that the evaluation of physiological status parameters such as GCS, sBP, and RR is challenging owing to their age-related variation and limited verbal communications/motor responses [21, 22]. There is a possibility of bias while evaluating the physiological status in younger pediatric patients and this may be reflected in the result of this study with a large rate of missing data of physiological status parameters in neonate/infant patients than the other age-groups (Table S1). Our results may suggest that a dataset with high-quality and without missing data may contribute to improving the accuracy of TRISS in predicting the survival of pediatric patients; however, improving the trauma database would arguably be difficult to achieve. Moreover, a previous study showed that RR data, which are missing in most cases in the Japan JTDB dataset, might be less needed for the calculation of TRISS Ps accuracy [9, 17, 23] and suggested that it may be effective to reduce the number of parameters or changes in the parameter in the prediction model for improving the accuracy of the model [24]. In the next step of research, therefore, not only modifying the coefficient of the TRISS model but also developing a new different prediction model that requires only easily collected and fewer missing data, may be necessary to improve the accuracy of survival prediction for pediatric trauma patients.

In the subclass analysis by TRISS Ps-interval groups, the accuracy of the performance of the TRISS model for patients with Ps ≤ 0.95 was low and the observed-to-expected mortality ratio in pediatric patients with Ps ≤ 0.25 was 2.15. Previous studies also suggested similar results, which are as follows: TRISS had lower performance in Japanese blunt trauma patients with Ps < 0.9 than those with Ps ≥ 0.9 [9] and TRISS underestimates survival for pediatric trauma patients with TRISS Ps ≤ 91% [10]. Previous studies suggested that the decreasing trend of in-hospital mortality among trauma patients decreased in recent years would lead the TRISS model to be out of calibration [8, 11]. Previous studies conducted using the JTDB data suggested that improvements in trauma care and trauma care systems account for decreasing mortality, especially in major trauma after the Japan Advanced Trauma Evaluation and Care was introduced in 2002 [7, 8, 25]. Therefore, our results may suggest that new coefficients related to injury severity should be calculated periodically to keep up with changes in trauma care in their own country.

Our study had several limitations. First, there was a selection bias because not all Japanese hospitals that treat have registered in the JTDB. Table S1 shows the rate of missing data by age category in the JTDB dataset. The number of neonates/infants with blunt trauma was lowest (*N* = 771, 2.9% of all), but the proportion of patients with missing data on survival and TRISS prediction was the largest (53.7% of neonate/infants with blunt trauma). These might have an adverse effect on the prediction accuracy of TRISS in neonates/infants. Therefore, a dataset with high-quality and without missing data should be constructed to improve the accuracy of TRISS in predicting the survival of pediatric patients. In addition, the number of participating hospitals differed across the study period. Furthermore, pediatric blunt trauma patients younger than 18 years whose data were registered in the JTDB (*N* = 7926, 30.1%) had missing data on important variables, although selection bias occurred in the data set with more than 10% missed rate [23]. Although this study population represents the Japanese trauma experience, our results may be nearly close to those obtained in many other Asian countries such as South Korea, Hong Kong, and Thailand, where trauma patient demographics are similar [12, 18, 26]. Our study attempted to utilize cross-validation procedures to assess the validity of the results obtained. In the next step, assessing the quality of the trauma care exactly by using the survival prediction model with higher accuracy than the current TRISS method could be achieved by using the data of each hospitals and type of trauma [1]. Therefore, developing a new regression model that is more suitable to the country’s situation, would result in better outcomes of trauma patients in that country, contributing to a decrease in the number of preventable trauma deaths.

## Conclusions

This study showed that overall the TRISS methodology appears to accurately predict survival in Japanese pediatric patients with blunt trauma. However, there were several problems in adopting the TRISS model for blunt trauma patients who are younger and/or with higher injury severity. In the future, it may be necessary to consider developing a simple, high-quality prediction model that is more suitable for pediatric trauma patients than the current TRISS model.

## Supplementary Information

**Additional file 1: Supplement 1.** Number of patients with missing data by age group and for each variable.

## Data Availability

The datasets supporting the conclusions of this article are available from the corresponding author on reasonable request.

## Abbreviations

TRISS: Trauma and Injury Severity Score
AUC: Area under the curve
Ps: Survival probability
MOTS: Major Trauma Outcome Study
JTDB: Japan Trauma Data Bank
AIS: Abbreviated Injury Scale
ISS: Injury Severity Score
GCS: Glasgow Coma scale
sBP: Systolic blood pressure
RR: Respiratory rate
ROC: Receiver operating characteristic
CI: 95% confidence interval
IQRs: Interquartile ranges

## References

[1] Lecky F, Woodford M, Edwards A, Bouamra O, Coats T (2014). Trauma scoring systems and databases. Br J Anaesth.

[2] Boyd CR, Tolson MA, Copes WS (1987). Evaluating trauma care: the TRISS method. Trauma score and the injury severity score. J Trauma.

[3] Champion HR, Sacco WJ, Copes WS, Gann DS, Gennarelli TA, Flanagan ME (1989). A revision of the trauma score. J Trauma.

[4] Champion HR, Copes WS, Sacco WJ, Lawnick MM, Keast SL, Bain LW (1990). The major trauma outcome study: establishing national norms for trauma care. J Trauma.

[5] Japan Advanced Trauma Evaluation and Care. http://www.jtcr-jatec.org/index_jatec.html (In Japanese). Accessed 20 July 2020.

[6] Japan Trauma Data Bank Report 2019 (2014–2018). https://www.jtcr-jatec.org/traumabank/dataroom/data/JTDB2019e.pdf. Accessed 20 July 2020.

[7] Hondo K, Shiraishi A, Fujie S, Saitoh D, Otomo Y (2013). In-hospital trauma mortality has decreased in Japan possibly due to trauma education. J Am Coll Surg.

[8] Nagata I, Abe T, Uchida M, Saitoh D, Tamiya N (2018). Ten-year inhospital mortality trends for patients with trauma in Japan: a multicentre observational study. BMJ Open.

[9] Suzuki T, Kimura A, Sasaki R, Uemura T (2017). A survival prediction logistic regression models for blunt trauma victims in Japan. Acute Med Surg.

[10] Schall LC, Potoka DA, Ford HR (2002). A new method for estimating the probability of survival in paediatric patients using the revised TRISS methodology based on age-adjusted weights. J Trauma.

[11] Rogers FB, Osler T, Krasne M, Rogers A, Bradburn EH, Lee JC (2012). Has TRISS become an anachronism? A comparison of mortality between the National Trauma Data Bank and major trauma outcome study databases. J Trauma Acute Care Surg.

[12] Chan CKO, Yau KKW, Cheung MT (2014). Trauma survival prediction in Asia population: a modification of TRISS to improve accuracy. Emerg Med J.

[13] Orliaguet G, Meyer P, Blanot S, Schmautz E, Charron B, Riou B (2001). Validity of applying TRISS analysis to paediatric blunt trauma patients managed in a French paediatric level I trauma Centre. Intensive Care Med.

[14] Ministry of Health Labour and Welfare. Vital Statistics/Vital statistics of Japan Final Data Classification table. http://www.mhlw.go.jp/english/database/db-hw/vs01.html. Accessed 6 Aug 2020.

[15] Association for the Advancement of Automotive Medicine (1998). The Abbreviated Injury Scale: 1990 Revision updated.

[16] Akobeng AK (2007). Understanding diagnostic tests 3: receiving operating characteristic curves. Acta Paediatr.

[17] Kimura A, Chadbunchachai W, Nakahara S (2012). Modification of the trauma and injury severity score (TRISS) method provides better survival prediction in Asian blunt trauma victims. World J Surg.

[18] Schluter PJ, Cameron CM, Davey TM, Civil I, Orchard J, Dansey R (2009). Contemporary New Zealand coefficients for the trauma injury severity score: TRISS(NZ). N Z Med J.

[19] Eichelberger MR, Champion HR, Sacco WJ, Gotschall CS, Copes WS, Bowman L.M. Pediatric coefficients for TRISS analysis. J Trauma 1993;34:319–322. doi: 10.1097/00005373-199303000-00001. 10.1097/00005373-199303000-00001 8483167

[20] Potoka DA, Schall LC, Ford HR (2001). Development of a novel age-specific pediatric trauma score. J Pediatr Surg.

[21] Nuttall AG, Paton KM, Kemp AM (2018). To what extent are GCS and AVPU equivalent to each other when assessing the level of consciousness of children with head injury? A cross-sectional study of UK hospital admissions. BMJ Open.

[22] Fleming S, Thompson M, Stevens R, Heneghan C, Plüddemann A, Maconochie I (2011). Normal ranges of heart rate and respiratory rate in children from birth to 18 years of age: a systematic review of observational studies. Lancet..

[23] Tohira H, Matsuoka T, Watanabe H, Ueno M (2011). Characteristics of missing data of the Japan trauma data Bank. JJAAM..

[24] Nakahara S, Ichikawa M, Kimura A (2011). Simplified alternative to the TRISS method for resource-constrained settings. World J Surg.

[25] Japan Advanced Trauma Evaluation and Care. Available online: http://www.jtcr-jatec.org/index_jatec.html (In Japanese). Accessed 20 July 2020.

[26] Kuwabara K, Matsuda S, Imanaka Y, Fushimi K, Hashimoto K, Ishikawa KB (2010). Injury severity score resource use and outcome for trauma patients within a Japanese administrative database. J Trauma.

